# Risk of colorectal cancer in first degree relatives of patients with colorectal polyps: nationwide case-control study in Sweden

**DOI:** 10.1136/bmj.n877

**Published:** 2021-05-04

**Authors:** Mingyang Song, Louise Emilsson, Bjorn Roelstraete, Jonas F Ludvigsson

**Affiliations:** 1 Departments of Epidemiology and Nutrition, Harvard T H Chan School of Public Health, Boston, MA, USA; 2 Clinical and Translational Epidemiology Unit, Massachusetts General Hospital and Harvard Medical School, Boston, MA, USA; 3 Division of Gastroenterology, Massachusetts General Hospital and Harvard Medical School, Boston, MA, USA; 4 Department of General Medicine, Institute of Health and Society, University of Oslo, Oslo, Norway; 5 Vårdcentralen Nysäter and Centre for Clinical Research, County Council of Värmland, Värmland, Sweden; 6 Department of Medical Epidemiology and Biostatistics, Karolinska Institute, 171 77 Stockholm, Sweden; 7 Faculty of Medicine and Health, Örebro University, Örebro, Sweden; 8 Department of Pediatrics, Örebro University Hospital, Örebro, Sweden; 9 Division of Digestive and Liver Disease, Department of Medicine, Columbia University Medical Center, New York, NY, USA; 10 Division of Epidemiology and Public Health, School of Medicine, University of Nottingham, Nottingham, UK

## Abstract

**Objective:**

To assess the risk of colorectal cancer (CRC) in first degree relatives (parents and full siblings) of patients with precursor lesions (polyps) for CRC.

**Design:**

Case-control study.

**Setting:**

Linkage to the multi-generation register and gastrointestinal ESPRESSO (Epidemiology Strengthened by histoPathology Reports in Sweden) histopathology cohort in Sweden.

**Participants:**

68 060 patients with CRC and 333 753 matched controls.

**Main outcome measures:**

Multivariable adjusted odds ratios of CRC according to the number of first degree relatives with a colorectal polyp and the histology of polyps and age at diagnosis in first degree relatives. Subgroup analysis was also performed according to age at CRC diagnosis and evaluated the joint association of family history of colorectal polyps and family history of CRC.

**Results:**

After adjusting for family history of CRC and other covariates, having a first degree relative with a colorectal polyp (8.4% (5742/68 060) in cases and 5.7% (18 860/333 753) in controls) was associated with a higher risk of CRC (odds ratio 1.40, 95% confidence interval 1.35 to 1.45). The odds ratios ranged from 1.23 for those with hyperplastic polyps to 1.44 for those with tubulovillous adenomas. To better put this risk in perspective, the age specific absolute risk of colon and rectal cancers was estimated according to family history of polyps based on the 2018 national CRC incidence in Sweden. For example, the absolute risk of colon cancer in individuals aged 60-64 years with and without a family history of colorectal polyp was, respectively, 94.3 and 67.9 per 100 000 for men and 89.1 and 64.1 per 100 000 for women. The association between family history of polyps and CRC risk was strengthened by the increasing number of first degree relatives with polyps (≥2 first degree relatives: 1.70, 1.52 to 1.90, P<0.001 for trend) and decreasing age at polyp diagnosis (<50 years: 1.77, 1.57 to 1.99, P<0.001 for trend). A particularly strong association was found for early onset CRC diagnosed before age 50 years (≥2 first degree relatives: 3.34, 2.05 to 5.43, P=0.002 for heterogeneity by age of CRC diagnosis). In the joint analysis, the odds ratio of CRC for individuals with two or more first degree relatives with polyps but no CRC was 1.79 (1.52 to 2.10), with one first degree relative with CRC but no polyps was 1.70 (1.65 to 1.76), and with two or more first degree relatives with both polyps and CRC was 5.00 (3.77 to 6.63) (P<0.001 for interaction).

**Conclusions:**

After adjusting for family history of CRC, the siblings and children of patients with colorectal polyps are still at higher risk of CRC, particularly early onset CRC. Early screening for CRC might be considered for first degree relatives of patients with polyps.

## Introduction

Colorectal cancer (CRC) is the second leading cause of cancer related deaths worldwide.^1^ Endoscopic screening reduces the incidence of and mortality from CRC by reoval of precursor lesions, namely colorectal polyps.^2^ ^3^ ^4^ Identifying high risk individuals for tailored screening has important public health and economic implications for improving the prevention of CRC.^5^ In contrast with the established increased risk associated with a family history of CRC, it remains unclear whether those with a family history of colorectal polyps have an increased risk of CRC.^6^ As a result, available screening recommendations are discrepant for individuals with a family history of polyps. The US Multi-Society Task Force on Colorectal Cancer recommends earlier screening in people with a family history of advanced polyps in the same manner as for those with a family history of CRC,^7^ whereas the British Society of Gastroenterology only considers family history of CRC but not polyps in screening recommendations.^8^ Given the higher prevalence of polyps than CRC associated with the increasing uptake of endoscopic screening, a better understanding about the influence of family history of polyps on CRC risk is critical to improve current screening recommendations.

Existing data on the association between family history of colorectal polyps and CRC risk are inconclusive. Whereas some studies reported an increased risk of CRC in first degree relatives of patients with a history of colorectal polyps,^9^ ^10^ ^11^ ^12^ others indicated that the risk varied by polyp type and was restricted to large or advanced adenomas.^13^ ^14^ Moreover, these studies had limited ability to inform screening recommendations owing to several limitations. Firstly, the most important unanswered question is whether the first degree relatives of someone with a colorectal polyp are still at greater risk of CRC after adjusting for family history of CRC. If the risk of CRC is increased, the next question is whether the first degree relatives of individuals with polyps are specifically at risk of early onset CRC that develops before the age of 50 years, the commonly recommended age for starting CRC screening in adults at average risk.^7^ ^8^ This is a particularly important question because of the global increase in the incidence of early onset CRC.^15^ In addition, previous studies have focused on conventional adenomas only, and the influence of family history of serrated polyps, a newly recognised precursor lesion for CRC, remains to be determined. Finally, past studies were limited by small sample size (<300 participants with CRC),^9^ ^11^ ^12^ ^13^ ^14^ cross sectional design,^11^ ^12^ ^13^ and reliance on self-reported family history data.^9^ ^11^ ^12^ ^13^ ^14^

In our nationwide case-control study, we used information from several national registers in Sweden to assess the relation between family history of colorectal polyps and risk of CRC.

## Methods

### Study population

We extracted data from several national registries in Sweden using the unique personal identity number assigned to all residents for the universal public healthcare system.^16^ Patients with CRC were obtained from the nationwide ESPRESSO (Epidemiology Strengthened by histoPathology Reports in Sweden) cohort, which included information on gastrointestinal biopsy samples from all 28 pathology departments in Sweden between 1965 and 2017.^17^ In ESPRESSO, histopathological findings were defined by morphology codes (a Swedish modification of the Systematized Nomenclature of Medicine (SNOMED) coding system) and topography codes. To identify patients with CRC, we used topography codes T67x (for colon) and T68x (for rectum) in combination with SNOMED codes (supplementary table 1). For each of the patients in ESPRESSO, we selected up to five controls from the general population matched on age at biopsy (in years), sex, year of birth, and county of residence.^17^

Supplementary figure 1 shows the flowchart of participant selection. For both cases and matched controls, we excluded people younger than 18 years, those with hereditary syndromes of CRC, and those with a history of inflammatory bowel disease and colectomy before CRC diagnosis. Because the multi-generation register is confined to individuals born after 1932, we further excluded those born before 1 January 1932. In total, 68 060 CRC cases and 333 753 matched controls were included in the study. Informed consent was waived by the Stockholm ethics review board because the study was strictly register based.^18^

### Assessment of family history of colorectal polyps and CRC

From the family relationship data in the Swedish multi-generation register,^19^ we retrieved information on family history and identified the parents and full siblings (first degree relatives) of our study participants. Then we assessed the history of colorectal polyps in the first degree relatives through linkage to the ESPRESSO cohort. Details of polyp assessment have been described previously.^20^ Briefly, we used topography codes T67/T68 in combination with SNOMED codes to identify the first diagnosis of colorectal polyps in first degree relatives. For conventional adenomas, SNOMED code M82100 was used for tubular adenomas, M82630 for tubulovillous adenomas, and M82611 for villous adenomas. Serrated polyps included hyperplastic polyps (M72040) and sessile serrated polyps. Given the evolving nature of the diagnosis criteria for sessile serrated polyps, we used a combination of SNOMED codes and free text search to identify such polyps. This approach has been validated with a positive predictive value of 93%.^21^

For each participant, we counted the number of first degree relatives with a polyp diagnosed before the index date, which was defined as the diagnosis date of CRC in cases and their matched controls. We also assessed the youngest age at polyp diagnosis and the histological subtypes of polyps among first degree relatives. Advanced polyps included tubulovillous or villous adenomas and sessile serrated polyps.

We assessed the history of CRC in first degree relatives based on the Cancer Register, which has recorded incident malignancies in Sweden since 1958 and has an estimated completeness of 96.3%.^22^ CRC was identified using ICD-7 (international classification of diseases, seventh revision) codes 153 and 154.

### Statistical analysis

Means (standard deviations) were calculated for continuous variables and percentages for categorical variables among cases and controls. We used conditional logistic regression to calculate the odds ratio and corresponding 95% confidence interval of CRC according to the history of colorectal polyps in first degree relatives (yes or no), the number of first degree relatives with a polyp (0, 1, ≥2), and the youngest age at polyp diagnosis in first degree relatives (<50, 50-59, 60-69, and ≥70 years). P values for trend were calculated by treating the number of relatives and age of diagnosis as a continuous variable. We considered three models: model 1 was conditional on the matching factors, and model 2 was further adjusted for other potential confounding factors (see supplementary methods for details of covariate assessment), including year of birth (continuous), family size (continuous), income levels (fifths), education (≤9 years, 10-12 years, >12 years, missing), total number of previous clinic visits (fifths), number of previous endoscopic examinations (0, 1, 2, and >2), Charlson comorbidity index score (continuous), and major comorbidities with a prevalence of at least 1% (all binary, including diabetes, cardiovascular disease, non-CRC, liver disease, chronic pulmonary disease, connective tissue disease, and peptic ulcer disease). To assess the effect of family history of polyps independent of family history of CRC, we further adjusted for the number of first degree relatives with a history of CRC (continuous) in the multivariable model (model 3).

To evaluate whether first degree relatives are specifically at risk for early onset CRC, we performed a subgroup analysis according to age at diagnosis of CRC (<50, 50-59, 60-69, and ≥70 years). We estimated the subgroup specific odds ratios using a fully unconstrained approach in which the confounder effects are allowed to be different among the CRC subgroups, and we used the contrast test method to calculate the P value for heterogeneity for the difference in the odds ratios across different age groups.^23^ The associations were also assessed according to specific subsites of CRC. Cancers from the caecum to transverse colon were classified as proximal colon cancer (topography codes T671-674), cancers from the splenic flexure to the sigmoid colon as distal colon cancer (T675-677), and those in the rectum or rectosigmoid junction as rectal cancer (T68x).

Furthermore, we performed stratified analysis according to sex, proband identity (parents, siblings), period of birth (<1940, 1940-49, ≥1950), and period of CRC diagnosis (<2008, 2008-11, ≥2012). In addition, we assessed the joint association of family history of polyps and family history of CRC with risk of overall CRC and early onset CRC. The P value for interaction was calculated using the Wald test for the product terms between the two joint factors.

To assess the influence of family history on absolute risk of CRC, we calculated the age specific CRC incidence in individuals with and without a family history of polyps based on the 2018 national CRC incidence in Sweden^24^ using the Greenland method for multivariate estimation of group specific incidence.^25^ ^26^ Because the national data are provided for colon and rectal cancer separately according to sex, our estimates also included each cancer subsite and sex.

Finally, to assess the robustness of our findings to ascertainment bias that might result from the earlier diagnosis of CRC in family members whose relatives had a history of polyps, we conducted a sensitivity analysis by using death from incident CRC as the outcome (that is, restricted to CRC cases who died from CRC). Furthermore, to better capture polyps diagnosed during routine screening, we also performed a sensitivity analysis by restricting to cases with CRC diagnosed after 2008, when organised screening started in the Stockholm region in Sweden among those aged 60-69 years using faecal occult blood testing,^27^ and by restricting the timeframe of family history assessment to the post-2008 period in Stockholm only. In addition, we assessed the number of diagnostic investigations performed in those with and without a family history of polyps by calculating the prevalence of common diseases diagnosed in the two groups. We used SAS 9.4 for the analyses. All statistical tests were two sided, with a P value of 0.05 considered to be significant.

### Patient and public involvement

Because we used national registers, patients and the public were not involved in the design or conduct of the study, or in the interpretation of the study results.

## Results

Table 1 shows the characteristics of the study participants. The mean age was 63 (SD 10) years and 46% of participants were women. Among cases, 7.7% (n=5220) and 0.8% (n=522) had one and two or more first degree relatives with a history of polyps, respectively, compared with 5.3% (n=17 627) and 0.4% (n=1233) in controls. The mean youngest age at polyp diagnosis in first degree relatives was 66.7 years for cases and 67.5 years for controls. Tubular adenomas were the most common polyp types in first degree relatives of both cases and controls, with a prevalence of 3.6% (n=2458) and 2.3% ((n=7783), respectively. Compared with controls, cases had a higher number of previous clinic visits and comorbidities.

**Table 1:** Characteristics of study participants. Values are numbers (percentages) unless stated otherwise

Characteristics	Cases (n=68 060)	Controls (n=333 753)
Women	31 464 (46.2)	154 526 (46.3)
Mean (SD) age (years)	63.4 (10.5)	63.3 (10.5)
Age groups (years):		
<50	7372 (10.8)	36 668 (11.0)
50-59	15 143 (22.3)	75 151 (22.5)
60-69	25 937 (38.1)	127 781 (38.3)
≥70	19 608 (28.8)	94 153 (28.2)
Mean (SD) year of birth	1943.3 (9.2)	1943.4 (9.2)
Mean (SD) No of FDRs	4.2 (2.6)	4.6 (3.0)
No of FDRs with polyps:		
0	62 318 (91.6)	314 893 (94.4)
1	5220 (7.7)	17 627 (5.3)
≥2	522 (0.8)	1233 (0.4)
No of FDRs with CRC:		
0	60 472 (88.9)	311 400 (93.3)
1	7059 (10.4)	21 446 (6.4)
≥2	529 (0.8)	907 (0.3)
Mean (SD) youngest age at polyp diagnosis in FDRs (years)*	66.7 (11.6)	67.5 (11.3)
Youngest age at polyp diagnosis in FDRs (years):		
<50	438 (0.1)	1170 (0.1)
50-59	1085 (0.2)	3464 (0.2)
60-69	1753 (0.3)	5724 (0.3)
≥70	2466 (0.4)	8502 (0.5)
Types of lesions in at least one FDR:		
Hyperplastic polyps	1667 (2.5)	6114 (1.8)
Sessile serrated polyps	123 (0.2)	437 (0.1)
Tubular adenomas	2458 (3.6)	7783 (2.3)
Tubulovillous adenomas	1856 (2.7)	5437 (1.6)
Villous adenomas	252 (0.4)	697 (0.2)
Advanced polyps	2194 (3.2)	6477 (1.9)
Mean (SD) total No of past clinic visits	10.4 (12.1)	7.4 (10.2)
Mean (SD) Charlson comorbidity index score	0.5 (0.9)	0.4 (0.8)
Comorbidities:		
Myocardial infarction	3178 (4.7)	13 694 (4.1)
Congestive heart failure	1926 (2.8)	6660 (2.0)
Peripheral vascular disease	1462 (2.2)	5371 (1.6)
Cerebrovascular disease	3952 (5.8)	16 936 (5.1)
Dementia	347 (0.5)	2283 (0.7)
Chronic pulmonary disease	4127 (6.1)	15 750 (4.7)
Connective tissue disease-rheumatic disease	1339 (2.0)	5842 (1.8)
Peptic ulcer disease	1872 (2.8)	3121 (0.9)
Mild liver disease	808 (1.2)	1798 (0.5)
Diabetes without complications	4663 (6.9)	14 414 (4.3)
Diabetes with complications	1654 (2.4)	5367 (1.6)
Paraplegia and hemiplegia	328 (0.5)	1262 (0.4)
Renal disease	629 (0.9)	2027 (0.6)
Cancer, excluding CRC	7422 (10.9)	25 802 (7.7)
Moderate or severe liver disease	213 (0.3)	534 (0.2)
Metastatic carcinoma of unspecified sites	1864 (2.7)	2098 (0.6)
AIDS/HIV	36 (0.1)	138 (0.0)

CRC=colorectal cancer; FDR=first degree relative (parents and full siblings).

All variables were assessed at time of CRC diagnosis for cases and their matched controls.

* Among FDRs with a history of polyps.

A history of polyps in first degree relatives was associated with a higher risk of CRC (multivariable odds ratio 1.62, 95% confidence interval 1.57 to 1.68; table 2). Further adjustment for family history of CRC led to a modest attenuation in the association (1.40, 1.35 to 1.45). For the rest of the results section, the odds ratios adjusted for family history of CRC are given. The odds ratios varied by polyp subtypes in first degree relatives, ranging from 1.23 (1.16 to 1.31) for hyperplastic polyps to 1.44 (1.36 to 1.53) for tubulovillous adenomas.

**Table 2:** Association between family history of polyps in first degree relatives (FDRs, parents and siblings) and risk of colorectal cancer (CRC). Values are numbers (percentages) unless stated otherwise

Polyp types in FDRs	Cases (n=68 060)	Controls (n=333 753)	Age adjusted odds ratio (95% CI)*	Multivariable adjusted odds ratio (95% CI)†	Multivariable+family history of CRC adjusted odds ratio (95% CI)‡
No polyps	62 318 (91.6)	314 893 (94.3)	1.00 (Ref)	1.00 (Ref)	1.00 (Ref)
Any polyp	5742 (8.4)	18 860 (5.7)	1.55 (1.50 to 1.60)	1.62 (1.57 to 1.68)	1.40 (1.35 to 1.45)
Advanced polyps	2194 (3.2)	6477 (1.9)	1.68 (1.60 to 1.77)	1.76 (1.67 to 1.86)	1.44 (1.36 to 1.51)
Serrated polyps:					
Hyperplastic	1667 (2.4)	6114 (1.8)	1.34 (1.27 to 1.42)	1.38 (1.30 to 1.46)	1.23 (1.16 to 1.31)
Sessile serrated	123 (0.2)	437 (0.1)	1.37 (1.12 to 1.67)	1.43 (1.16 to 1.77)	1.27 (1.03 to 1.57)
Conventional adenomas:					
Tubular	2458 (3.6)	7783 (2.3)	1.57 (1.50 to 1.64)	1.62 (1.54 to 1.70)	1.39 (1.32 to 1.46)
Tubulovillous	1856 (2.7)	5437 (1.6)	1.69 (1.60 to 1.79)	1.77 (1.67 to 1.87)	1.44 (1.36 to 1.53)
Villous	252 (0.4)	697 (0.2)	1.77 (1.54 to 2.05)	1.82 (1.57 to 2.12)	1.40 (1.20 to 1.63)

* Conditional logistic regression was used to account for matching on age, sex, year of birth, and county of residence.

† Multivariable model was further adjusted for year of birth (continuous), family size (continuous), income levels (fifths), education (≤9 years, 10-12 years, >12 years, missing), total number of previous clinic visits (fifths), number of previous endoscopies (0, 1, 2, ≥3), Charlson comorbidity index score (continuous), and major comorbidities (all binary, including diabetes, cardiovascular disease, non-colorectal cancer, liver disease, chronic pulmonary disease, connective tissue disease, and peptic ulcer disease).

‡ Further adjusted for family history of CRC in FDRs.

The association between family history of polyps and risk of CRC strengthened with the increasing number of first degree relatives with polyps (odds ratio for ≥2 first degree relatives with polyps: 1.70, 1.52 to 1.90, P<0.001 for trend) and decreasing age at polyp diagnosis (<50 years, 1.77, 1.57 to 1.99, P<0.001 for trend; table 3). Similar patterns were found for individual polyp subtypes (see supplementary tables 2 and 3). When the joint association of the number of first degree relatives with a history of polyps and youngest age at polyp diagnosis was examined (table 4), the odds ratios ranged from 1.33 (1.28 to 1.39) for one first degree relative and youngest age at polyp diagnosis 60 years or older, to 1.82 (1.49 to 2.22) for two or more first degree relatives and youngest age at polyp diagnosis less than 60 years.

**Table 3:** Risk of colorectal cancer (CRC) according to number of first degree relatives (FDRs, parents and siblings) with a family history of polyps. Values are numbers (percentages) unless stated otherwise

Polyp types in FDRs	Cases (n=68 060)	Controls (n=333 753)	Multivariable adjusted odds ratio (95% CI)*	Multivariable+family history of CRC adjusted odds ratio (95% CI)†
**Any polyps**				
No of FDRs with any polyps:				
0	62 318 (91.6)	314 893 (94.3)	1 (Ref)	1 (Ref)
1	5220 (7.7)	17 627 (5.3)	1.58 (1.53 to 1.63)	1.38 (1.33 to 1.43)
≥2	522 (0.8)	1233 (0.4)	2.27 (2.03 to 2.53)	1.70 (1.52 to 1.90)
P for trend	—	—	<0.001	<0.001
Youngest age at diagnosis of any polyps (years):				
≥70	2466 (3.6)	8502 (2.5)	1.52 (1.45 to 1.60)	1.29 (1.23 to 1.36)
60-69	1753 (2.6)	5724 (1.7)	1.66 (1.57 to 1.76)	1.44 (1.35 to 1.52)
50-59	1085 (1.6)	3464 (1.0)	1.67 (1.56 to 1.80)	1.48 (1.37 to 1.59)
<50	438 (0.6)	1170 (0.4)	2.00 (1.78 to 2.24)	1.77 (1.57 to 1.99)
P for trend	—	—	<0.001	<0.001
**Advanced polyps**				
No of FDRs with advanced polyps:				
0	65 866 (96.8)	327 276 (98.1)	1 (Ref)	1 (Ref)
1	2137 (3.1)	6326 (1.9)	1.76 (1.67 to 1.85)	1.44 (1.36 to 1.52)
≥2	57 (0.1)	151 (0.0)	1.93 (1.41 to 2.66)	1.26 (0.91 to 1.75)
P for trend	—	—	<0.001	<0.001
Youngest diagnosis age of advanced polyps (years):				
≥70	1067 (1.6)	3451 (1.0)	1.59 (1.48 to 1.71)	1.29 (1.20 to 1.39)
60-69	638 (0.9)	1785 (0.5)	1.87 (1.70 to 2.06)	1.51 (1.37 to 1.67)
50-59	371 (0.5)	989 (0.3)	1.98 (1.74 to 2.24)	1.64 (1.45 to 1.87)
<50	118 (0.2)	252 (0.1)	2.57 (2.04 to 3.23)	2.12 (1.68 to 2.68)
P for trend	—	—	<0.001	<0.001

* Multivariable conditional logistic regression was used to account for matching on age, sex, year of birth, and county of residence, and was further adjusted for year of birth (continuous), family size (continuous), income levels (fifths), education (≤9 years, 10-12 years, >12 years, missing), total number of previous clinic visits (fifths), number of previous endoscopies (0, 1, 2, ≥3), Charlson comorbidity index score (continuous), and major comorbidities (all binary, including diabetes, cardiovascular disease, non-colorectal cancer, liver disease, chronic pulmonary disease, connective tissue disease, and peptic ulcer disease).

† Further adjusted for number of FDRs with a history of CRC (continuous).

**Table 4:** Risk of total and age specific colorectal cancer (CRC) according to number of first degree relatives (FDRs, parents and siblings) with polyps and youngest age at polyp diagnosis. Vales are numbers (percentages) unless stated otherwise

Family history of polyps	Cases (n=68 060)	Controls (n=333 753)	Multivariable adjusted odds ratio (95% CI)*	Multivariable+family history of CRC adjusted odds ratio (95% CI)†	P value
**Any polyps**					
Any FDR, youngest age at diagnosis <60 years	1523 (2.4)	4634 (1.5)	1.75 (1.64 to 1.86)	1.53 (1.44 to 1.64)	<0.001
1 FDR, youngest age at diagnosis ≥60 years	3880 (5.9)	13 367 (4.1)	1.54 (1.48 to 1.61)	1.33 (1.28 to 1.39)	<0.001
1 FDR, youngest age at diagnosis <60 years	1340 (2.1)	4260 (1.3)	1.68 (1.57 to 1.80)	1.51 (1.41 to 1.61)	<0.001
≥2 FDRs, any age	522 (0.8)	1233 (0.4)	2.23 (1.99 to 2.49)	1.64 (1.46 to 1.84)	<0.001
≥2 FDRs, youngest age at diagnosis <60 years	183 (0.3)	374 (0.1)	2.51 (2.07 to 3.04)	1.82 (1.49 to 2.22)	<0.001
**Advanced polyps**					
Any FDR, youngest age at diagnosis <60 years	489 (0.7)	1241 (0.4)	2.10 (1.88 to 2.35)	1.73 (1.54 to 1.94)	<0.001
1 FDR, youngest age at diagnosis ≥60 years	1660 (2.5)	5129 (1.5)	1.67 (1.57 to 1.77)	1.36 (1.28 to 1.45)	<0.001
1 FDR, youngest age at diagnosis <60 years	477 (0.7)	1197 (0.4)	2.14 (1.91 to 2.39)	1.77 (1.58 to 1.99)	<0.001
≥2 FDRs, any age	57 (0.1)	151 (0.0)	1.92 (1.39 to 2.65)	1.22 (0.88 to 1.70)	0.24
≥2 FDRs, youngest age at diagnosis <60 years	12 (0.0)	44 (0.0)	1.21 (0.62 to 2.38)	0.73 (0.36 to 1.47)	0.38

* Multivariable conditional logistic regression was used to account for the matching on age, sex, year of birth, and county of residence, and was further adjusted for year of birth (continuous), family size (continuous), income levels (fifths), education (≤9, 10-12 years, >12 years, missing), total number of previous clinic visits (fifths), number of previous endoscopies (0, 1, 2, ≥3), Charlson comorbidity index score (continuous), and major comorbidities (all binary, including diabetes, cardiovascular disease, non-colorectal cancer, liver disease, chronic pulmonary disease, connective tissue disease, and peptic ulcer disease).

† Further adjusted for number of FDRs with a history of CRC (continuous).

**Fig 1:**
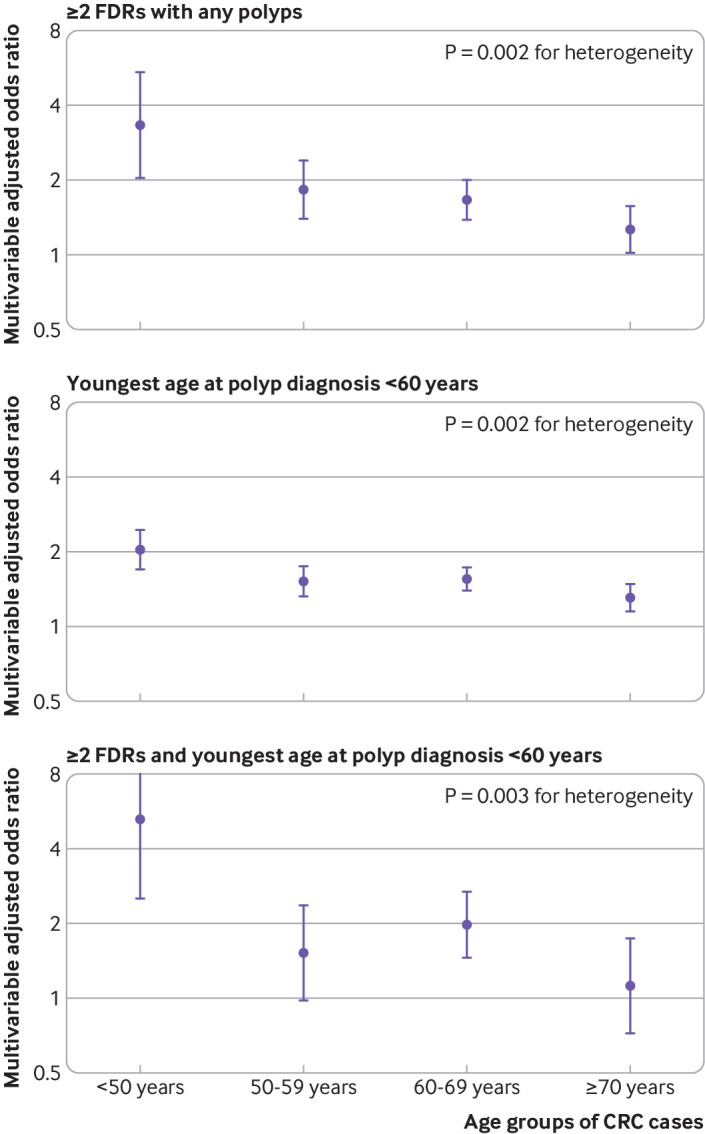
Risk of colorectal cancer (CRC) according to age at diagnosis in relation to number of first degree relatives (FDRs) with any polyps and youngest age at polyp diagnosis. Odds ratios are presented on the log(2) scale and were calculated using the fully adjusted conditional logistic regression model that included family history of CRC

When the risk of CRC was investigated according to age at diagnosis (fig 1), the association between family history of polyps and risk of CRC declined with age (P<0.01 for heterogeneity). A particularly strong association was found for early onset CRC diagnosed before age 50 years, with an odds ratio of 3.34 (2.05 to 5.43) for two or more first degree relatives with polyps, 2.03 (1.69 to 2.43) for the youngest age at polyp diagnosis in first degree relatives of less than 60 years, and 5.27 (2.54 to 10.91) for having two or more first degree relatives with polyps along with youngest age at polyp diagnosis of less than 60 years.

When assessed by CRC subsites, no statistically significant difference was found for a family history of any polyps, conventional adenomas, or serrated polyps, although a family history of serrated polyps seemed to be strongly associated with risk of proximal colon cancer (odds ratio for ≥2 first degree relatives: 2.53, 1.06 to 6.03) but not distal colon cancer (0.96, 0.37 to 2.46) or rectal cancer (1.14, 0.68 to 1.92; see supplementary table 4).

Stratified analysis did not reveal any substantial difference in the association of family history of polyps with risk of CRC according to sex or proband identity, whereas a stronger association was found for more recent birth cohort (P=0.01 for interaction) and for CRCs diagnosed before 2008 (P=0.003 for heterogeneity; see supplementary table 5).

**Fig 2:**
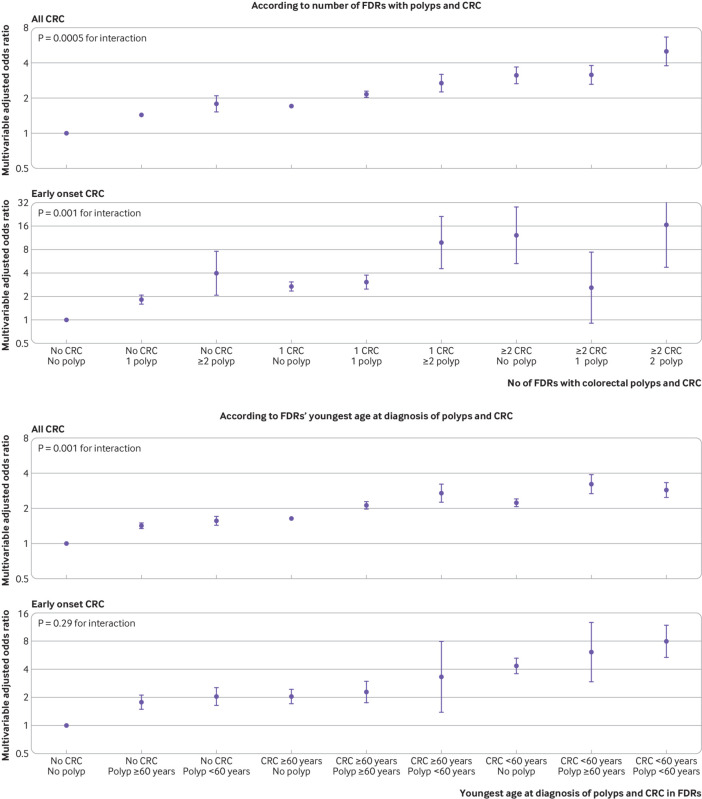
Joint association of history of polyps and colorectal cancer (CRC) in first degree relatives (FDRs) with risk of overall CRC and early onset CRC (diagnosed before age 50 years). Odds ratios are presented on the log(2) scale and were calculated using the fully adjusted conditional logistic regression model. See supplementary table 6 for detailed results

Figure 2 presents the joint association of family history of polyps and family history of CRC. Even among participants without a family history of CRC (cases: n=60 472 (88.9%); controls: n=311 400 (93.3%)), those with a family history of polyps (cases: n=3475 (5.7%); controls: n=13 303 (4.3%)) still showed a higher risk of CRC. Compared with individuals with no first degree relatives with either polyps or CRC, the odds ratio for those having two or more first degree relatives with polyps but no CRC was 1.79 (1.52 to 2.10), for those having one first degree relative with CRC but no polyps was 1.70 (1.65 to 1.76), and for those having two or more first degree relatives with both polyps and CRC was 5.00 (3.77 to 6.63) (P<0.001 for interaction). A similar synergistic association was found when individuals were classified based on age at diagnosis of polyps and CRC in their first degree relatives (P=0.001 for interaction); and those with first degree relatives in whom both polyps and CRC had been diagnosed before age 60 years had an OR of 2.85 (2.48 to 3.29). A more noticeable association was found for early onset CRC, with an odds ratio of 16.57 (4.81 to 57.13) for individuals having two or more first degree relatives with both polyps and CRC (see supplementary table 6 for detailed results).

**Fig 3:**
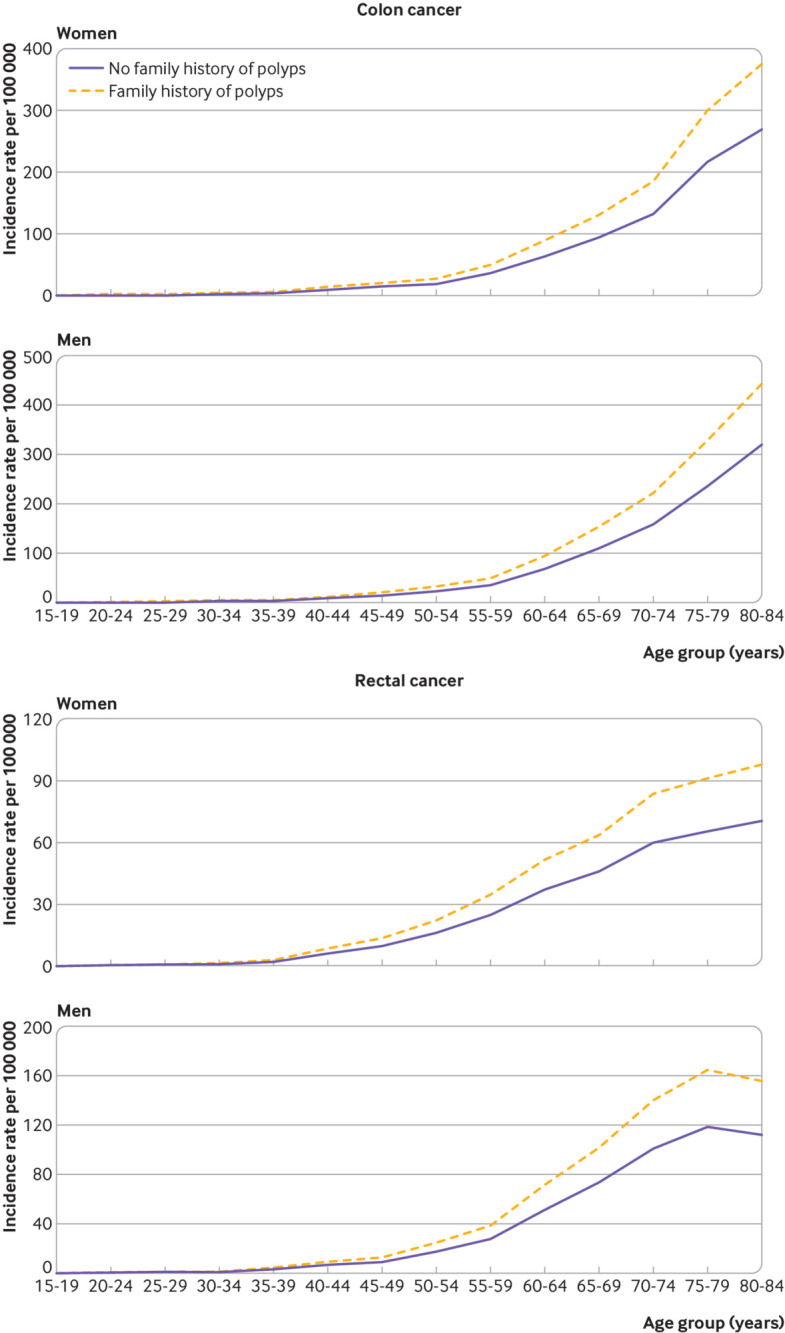
Estimated absolute incidence of colon cancer and rectal cancer in women and men in those with and without a family history of polyps, according to age

Figure 3 shows the age specific absolute incidence of CRC according to family history of polyps. As CRC incidence increases with age, the risk difference between those with and those without a family history of polyps also showed a substantial increase. For example, the risk difference of colon cancer in men showed an increase from 5.6 per 100 000 in those aged 45-49 years to 124.2 per 100 000 in those aged 80-84 years.

To investigate the potential influence of ascertainment bias, the mortality from incident CRC was assessed as the outcome in a sensitivity analysis. A family history of polyps was found to be similarly associated with mortality of incident CRC (multivariable odds ratio 1.40, 95% confidence interval 1.30 to 1.51; see supplementary table 7). The sensitivity analyses restricted to cases of CRC diagnosed after 2008 also showed similar results (see supplementary table 7). When the prevalence of common diseases was compared, no substantial differences were observed in those with and those without a family history of polyps (see supplementary table 8). Taken together, these results indicate little influence of ascertainment bias.

## Discussion

In this nationwide case-control study, using data from established national registers in Sweden, individuals with a history of CRC precursor lesions (ie, polyps) in first degree relatives were shown to have a 62% increased risk of CRC compared with those with no family history of polyps. The increase in risk was attenuated to 40% after adjusting for family history of CRC but increased to 70-77% when more than one first degree relative had a polyp or when a polyp was first diagnosed in a first degree relative before age 50 years. A particularly strong association was found for early onset CRC. In the joint analysis of family history of polyps and CRC, individuals who had two or more first degree relatives with polyps but no CRC were found to be at a slightly higher risk of CRC compared with those who had one first degree relative with CRC but no polyps; and those who had two or more first degree relatives with both polyps and CRC had a fivefold increased risk of overall CRC and more than a 16-fold increased risk of early onset CRC, compared with individuals with no first degree relatives with polyps or CRC. These findings provide robust evidence for the impact of family history of polyps on risk of CRC and have important implications for CRC screening.

### Comparison with other studies and implications

Our study extends knowledge in several respects. Firstly, we specifically examined the independent and joint associations of history of polyps and CRC in first degree relatives with risk of CRC. Without mutual adjustment, our effect estimates are consistent with those in previous studies, with the odds ratio of 1.62 for one first degree relative or more with polyps (range 1.35-1.78 in past studies)^9^ ^10^ ^11^ ^14^ and 1.83 for one first degree relative or more with CRC (range 1.80-2.25 in past studies).^6^ After mutual adjustment, a family history of polyps was still associated with a 40% increased risk of CRC. Moreover, in a joint analysis we found a synergistic association between a family history of polyps and CRC. First degree relatives with a history of both conditions had a substantially increased risk of CRC compared with those without either. A higher risk was found in individuals with two more first degree relatives with polyps but no CRC (odds ratio 1.79) than in those with one first degree relative with CRC but no polyps (odds ratio 1.70). Taken together, these findings indicate that a family history of polyps might provide critical additional information beyond a family history of CRC for risk assessment in first degree relatives. Individuals with at least two first degree relatives with polyps, most of whom are not yet recommended for early screening according to existing recommendations,^7^ ^8^ might benefit from and be considered for early CRC screening as those with one first degree relative with CRC.

Secondly, we addressed another yet unanswered critical question about how a family history of polyps might be associated with risk of CRC according to age of onset in first degree relatives. We found that the association tended to increase with decreasing age at CRC diagnosis, with a particularly strong association observed for early onset CRC. These data have critical implications for refining the current screening recommendations. Although screening in the US has contributed substantially to the decreasing incidence of CRC in older adults,^28^ incidence has increased by 0.5-2.4% annually since the mid-1980s in adults younger than 50 years, for whom routine screening has not been recommended.^29^ A similar increase has been documented in many other regions worldwide.^15^ This trend led the American Cancer Society to lower its recommended age for starting CRC screening from 50 to 45 years.^30^ This revision has led to intense debate about the potential benefits, harms, liabilities, and costs.^31^ ^32^ ^33^ ^34^ ^35^ ^36^ Recently, the US Preventive Services Task Force issued a draft recommendation that also lowered the starting age of CRC screening to 45 years. Since the incidence of early onset CRC remains much lower than that of late onset CRC, a risk based approach for screening might be particularly appealing for younger adults. Our observation for a strong association between family history of polyps and early onset CRC suggests that an assessment of family history might be considered to tailor screening for better prevention of early onset CRC.

Thirdly, we performed detailed analysis according to histological subtype of polyps in first degree relatives. In line with the increasing recognition of the role of serrated polyps in CRC, we found that first degree relatives of patients with serrated polyps had an increased risk of CRC and that the increased risk was similar to that of patients with conventional adenomas. Moreover, in support of the predominant effect of the serrated pathway on development of proximal colon cancer,^37^ we observed a stronger association of family history of serrated polyps with proximal colon cancer. These findings suggest a familial clustering of neoplastic pathways in CRC and have implications for studying hereditary risk of CRC. Interestingly, we found that a family history of hyperplastic polyps was also associated with a moderately increased risk of CRC. This is consistent with our previous findings in the ESPRESSO cohort for an increased risk of CRC associated with hyperplastic polyps, possibly as a result of the misdiagnosis of sessile serrated polyps as hyperplastic polyps.^20^ However, given the evolving diagnosis criteria of serrated polyps,^38^ our findings should be interpreted with caution. In addition, compared with first degree relatives of patients with any polyps, the increase in risk of CRC in those with polyps of advanced histology was only slightly higher. In contrast, the increasing number of first degree relatives with polyps, and, to a lesser extent, the younger age at polyp diagnosis seemed to have a stronger influence on risk of CRC. These results are not unexpected because compared with polyp histology in first degree relatives, a higher number of relatives with polyps and the early onset of polyps might better reflect the familial risk associated with genetic susceptibility and early environmental factors. These findings suggest that the information on polyp histology in first degree relatives might not be as critical as currently perceived for CRC risk assessment. As a result, the concern about lack of accurate recall or documentation of polyp histology could be eased when making screening recommendations based on family history of polyps.^39^

Finally, we found that proband identity (parents and siblings) did not affect the risk of CRC associated with family history of polyps. This is consistent with previous data indicating a similar risk of colorectal neoplasia among individuals with different first degree relatives affected with CRC.^40^

### Strengths and limitations of this study

Our study has several strengths, including the nationwide population based design; large sample size; high validity of the cancer and polyp registers; comprehensive assessment of the number of first degree relatives, their age at diagnosis, and histological subtypes of polyps; and detailed subgroup analysis according to CRC subsites and age of onset. In particular, our use of objective register based data minimises measurement error in family history assessment and represents a major advantage over previous studies that relied on patients’ recall.^9^ ^11^ ^12^ ^13^ ^14^

Several limitations of our study also need to be noted. Firstly, we lacked information on other factors that might influence the risk of CRC, including the size and multiplicity of polyps in first degree relatives and lifestyle risk factors. However, we have established the predictivity of different histological polyp subtypes for CRC risk in the study population.^20^ Moreover, we adjusted for the Charlson comorbidity index score and several major individual comorbidities that are strongly associated with CRC risk factors (eg, smoking and obesity). Secondly, the nationwide polyp data in ESPRESSO are confined to 1965 and onwards, with limited coverage before 1990. As a result, we might have missed some first degree relatives with polyps diagnosed in earlier years, leading to misclassification of some individuals with a family history of polyps that might have biased our results towards the null. Thirdly, we lacked information on indications of endoscopic examination for polyp detection in first degree relatives. As organised screening did not start regionally in Sweden until 2008, the polyps in first degree relatives of our study participants are likely to be clinically detected (ie, based on symptoms) with a low prevalence and our findings might not be generalisable to populations in which screening endoscopy is common. However, any misclassification in the polyp history in first degree relatives is unlikely to differ between the index cases and controls and thus could only have attenuated our effect estimates. Indeed, we observed a stronger association among individuals in the more recent birth cohort, for whom misclassification was reduced and who had a greater family history of polyps. Also, the sensitivity analysis of restricting the timeframe of family history assessment to the post-2008 period in Stockholm and CRC cases diagnosed after 2008 revealed similar results, indicating the robustness of our findings.

### Conclusions

After adjusting for family history of CRC, siblings and children of patients with colorectal polyps were found to be at increased risk of CRC, particularly when polyps were diagnosed in more than one first degree relative or before age 60 years. The increased risk is more prominent for early onset CRC and heightened in association with a family history of CRC. Our findings suggest that to better prevent early onset CRC, early screening, if proved effective, might be tailored for first degree relatives of individuals with polyps, particularly those with multiple first degree relatives with a history of polyps and when polyps are diagnosed in first degree relatives at a younger age.

**What is already known on this topic**

Endoscopic screening reduces the incidence of and mortality from colorectal cancer (CRC) by removal of precursor lesions—namely, colorectal polyps
A family history of CRC is an established risk factor for CRC
Individuals with a history of advanced colorectal polyps are at higher risk of developing CRC

**What this study adds**

Individuals with at least two first degree relatives with polyps or a first degree relative with polyps diagnosed at a young age, most of whom are not yet recommended for early screening according to existing guidelines, are at an increased risk of CRC, particularly early onset disease, and they might benefit from early screening
Compared with the advanced histology of polyps, the higher number of first degree relatives with polyps and younger age at polyp diagnosis seemed to be more predictive of CRC risk in family members

## Web extra

Extra material supplied by authors

Web appendix: Supplementary materials

## Notes

Contributors: MS and LE conceived and designed the study. JFL acquired the data. MS had access to all the data, performed all analyses, and designed the figures. MS and JFL obtained funding. MS wrote the first draft of the manuscript. All authors critically revised the manuscript for important intellectual content and approved the final version. JFL supervised the study. MS is the guarantor. The corresponding author attests that all listed authors meet authorship criteria and that no others meeting the criteria have been omitted.

Ethical approval: This study was approved by the Stockholm ethical review board (2014/1287-31/4).

Data sharing: No additional data available.

The lead author (MS) affirms that this manuscript is an honest, accurate, and transparent account of the study being reported; that no important aspects of the study have been omitted; and that any discrepancies from the study as planned (and, if relevant, registered) have been explained.

Dissemination to participants and related patient and public communities: We plan to disseminate the results of our research to the public and relevant patient community through the current publication and its related press release as well as presentations at scientific conferences.

Provenance and peer review: Not commissioned; externally peer reviewed.
