# Supplementary material for: Risk of colorectal cancer in first degree relatives of patients with colorectal polyps: nationwide case-control study in Sweden
Source: BMJ. 2021 May 4;373:n877. doi: 10.1136/bmj.n877 (PMC8083372; doi:10.1136/bmj.n877)
Supplement: Supplementary file 1 — Web appendix: Supplementary materials [file sonm063617.ww.pdf]

Supplementary Materials

Supplementary Methods - Assessment of covariates

Supplementary Figure 1. Flowchart of participant selection

Supplementary Table 1. SNOMED codes used to identify patients with colorectal cancer

Supplementary Table 2. Risk of colorectal cancer according to number of FDRs with a positive history of different histological subtypes of polyps

Supplementary Table 3. Risk of colorectal cancer according to the youngest age of diagnosis in FDRs for different histological subtypes of polyps

Supplementary Table 4. Association between family history of polyps in FDRs and risk of colorectal cancer according to cancer subsites

Supplementary Table 5. Stratified association between family history of any polyps in FDRs and risk of colorectal cancer

Supplementary Table 6. Joint association of history of polyps and colorectal cancer (CRC) in first-degree relatives (FDRs) with risk of overall CRC and early-onset CRC (diagnosed before the age of 50 years).

Supplementary Table 7. Summary of results of sensitivity analyses for assessing the association between family history of any polyps and risk of colorectal cancer (CRC)

Supplementary Table 8. Prevalence of common diseases diagnosed in individuals with and without a family history of polyps among the control group

Supplementary Methods

Assessment of covariates

For each participant, we calculated the family size as the sum of the number of parents and siblings. We obtained data on education and income from the longitudinal integrated database for health insurance and labor market studies,[1] which integrates annually updated administrative information from the labor market and educational and social sectors from 1990 onward on all individuals 16 years or older registered as residents in Sweden. We used the education and income data closest to the time of CRC diagnosis for both cases and their matched controls. Information on age, sex, date of birth, and emigration status was collected form the Swedish Total Population Register maintained by Statistics Sweden.

We collected information on use of endoscopic examination from the Swedish National Patient Registry, which started in 1964 with complete national coverage from 1987. We used the established procedure codes to identify colonoscopy (9011, 9023, 4688, 4689, 4674, 4684, UJF32, and UJF35) and sigmoidoscopy (9012, 4685, UJF42, and UJF45).[2] We counted the number of endoscopies performed before CRC diagnosis for cases and their matched controls. To avoid counting the diagnostic endoscopies for CRC, we excluded endoscopies performed within 30 days before the date of CRC diagnosis. We calculated the Charlson comorbidity score using the SAS® macro code developed by Turner and Burchill[3] based on the established ICD-9 and ICD-10 coding algorithms for 17 comorbidities,[4 5] including diabetes that has been linked to higher CRC risk. We also calculated the total number of prior clinic visits before CRC diagnosis for cases and their matched controls using the inpatient and outpatient records.

References

1. Ludvigsson JF, Svedberg P, Olen O, et al. The longitudinal integrated database for health insurance and labour market studies (LISA) and its use in medical research. *Eur J Epidemiol* 2019;34(4):423-37. doi: 10.1007/s10654-019-00511-8 [published Online First: 2019/04/01]
2. Ludvigsson JF, Lebwohl B, Ekbom A, et al. Outcomes of Pregnancies for Women Undergoing Endoscopy While They Were Pregnant: A Nationwide Cohort Study. *Gastroenterology* 2017;152(3):554-63 e9. doi: 10.1053/j.gastro.2016.10.016 [published Online First: 2016/10/25]
3. Turner K, Burchill C. SAS® macro code for %CharlsonICD10 2006 [Available from: http://mchp-appserv.cpe.umanitoba.ca/Upload/SAS/_CharlsonICD10.sas.txt.
4. Quan H, Sundararajan V, Halfon P, et al. Coding algorithms for defining comorbidities in ICD-9-CM and ICD-10 administrative data. *Med Care* 2005;43(11):1130-9. doi: 10.1097/01.mlr.0000182534.19832.83 [published Online First: 2005/10/15]
5. Charlson ME, Pompei P, Ales KL, et al. A new method of classifying prognostic comorbidity in longitudinal studies: development and validation. *J Chronic Dis* 1987;40(5):373-83. doi: 10.1016/0021-9681(87)90171-8 [published Online First: 1987/01/01]

Supplementary Figure 1. Flowchart of participant selection. Abbreviations: ESPRESSO, Epidemiology Strengthened by histoPathology Reports in Sweden.

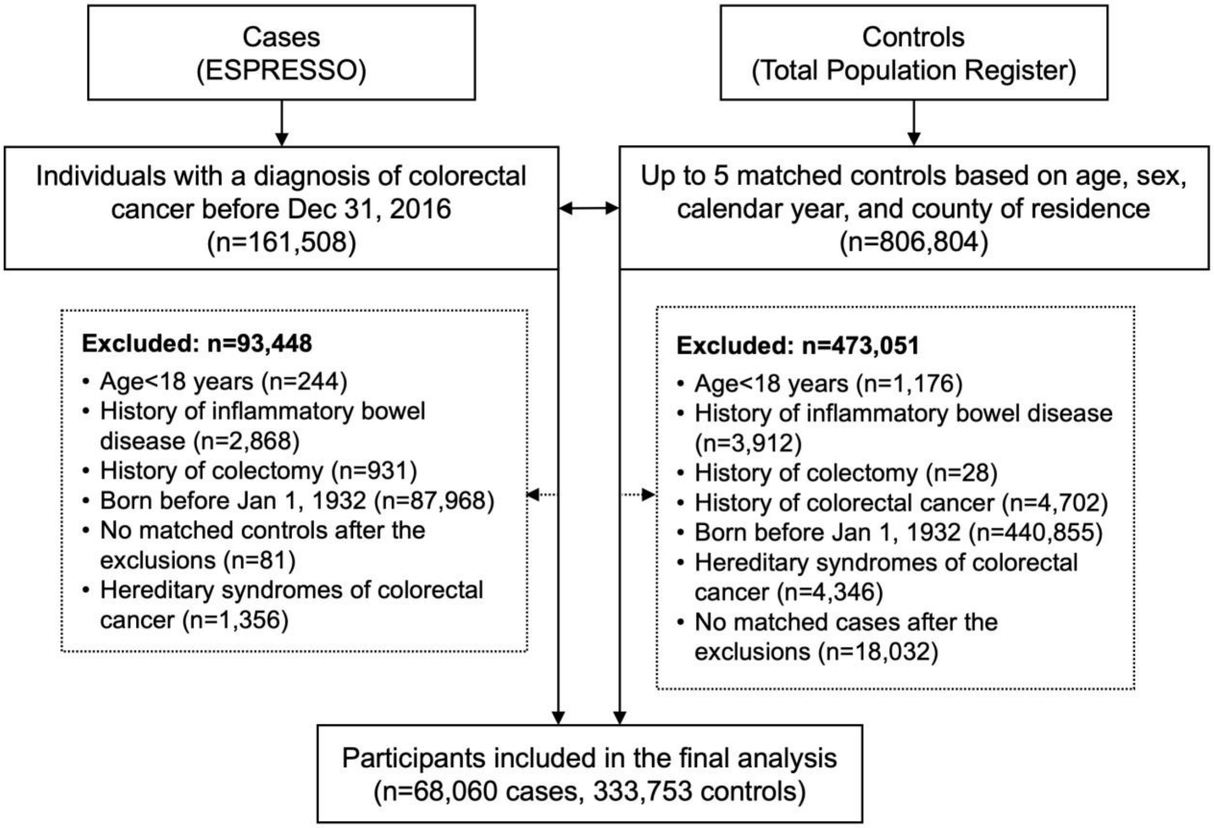

Supplementary Table 1. SNOMED codes used to identify patients with colorectal cancer (CRC)

SNOMED code	Description
M81401	Adenocarcinoma - suspected
M81402	Adenocarcinoma - in situ
M81403	Adenocarcinoma
M81404	Adenocarcinoma, overgrowth
M81406	Metastasis from adenocarcinoma
M81407	Recurrence of adenocarcinoma
M81409	Primary adenocarcinoma
M81423	Colonic linitis plastica
M81443	Adenocarcinoma - intestinal type
M81453	Adenocarcinoma - diffuse type

Supplementary Table 2. Risk of colorectal cancer according to number of FDRs with a positive history of different histological subtypes of polyps

Polyp types in FDRs	0	1	≥2	P_{trend}
Hyperplastic polyps				
No. of cases (%)	66,393 (97.6)	1,606 (2.4)	61 (0.1)	
No. of controls (%)	327,639 (98.2)	5,922 (1.8)	192 (0.1)	
Multivariable-adjusted OR (95% CI)*	1 (Ref)	1.23 (1.16-1.31)	1.22 (0.90-1.67)	<.0001
Sessile serrated polyps				
No. of cases (%)	67,937 (99.8)	123 (0.2)	0 (0.0)	
No. of controls (%)	333,316 (99.9)	437 (0.1)	0 (0.0)	
Multivariable-adjusted OR (95% CI)*	1 (Ref)	1.27 (1.03-1.57)	-	0.03
Tubular adenomas				
No. of cases (%)	65,602 (96.4)	2,332 (3.4)	126 (0.2)	
No. of controls (%)	325,970 (97.7)	7,531 (2.3)	252 (0.1)	
Multivariable-adjusted OR (95% CI)*	1 (Ref)	1.37 (1.31-1.45)	1.89 (1.50-2.38)	<.0001
Tubulovillous adenomas				
No. of cases (%)	66,204 (97.3)	1,814 (2.7)	42 (0.1)	
No. of controls (%)	328,316 (98.4)	5,324 (1.6)	113 (0.0)	
Multivariable-adjusted OR (95% CI)*	1 (Ref)	1.45 (1.36-1.53)	1.16 (0.79-1.69)	<.0001
Villous adenomas				
No. of cases (%)	67,808 (99.6)	251 (0.4)	1 (0.0)	
No. of controls (%)	333,056 (99.8)	696 (0.2)	1 (0.0)	
Multivariable-adjusted OR (95% CI)*	1 (Ref)	1.40 (1.20-1.63)	1.48 (0.09-23.75)	<.0001

Abbreviations: CI, confidence interval; FDR, first-degree relative; OR, odds ratio.

*Multivariable conditional logistic regression was used to account for the matching on age, sex, year of birth, and county of residence, and was further adjusted for the number of FDRs with a history of CRC (continuous), year of birth (continuous), family size (continuous), income levels (quintiles), education (9 years or less, 10-12 years, >12 years, missing), total number of prior clinic visits (quintiles), number of prior endoscopies (0, 1, 2, ≥3), Charlson comorbidity score (continuous), and major comorbidities (all binary, including diabetes, cardiovascular disease, non-colorectal cancer, liver disease, chronic pulmonary disease, connective tissue disease, and peptic ulcer disease).

Supplementary Table 3. Risk of colorectal cancer according to the youngest age of diagnosis in FDRs for different histological subtypes of polyps

Polyp types in FDRs	≥70 years	60-69 years	50-59 years	<50 years	P_{trend}
Hyperplastic polyps					
No. of cases (%)	503 (0.7)	572 (0.8)	427 (0.6)	165 (0.2)	
No. of controls (%)	1,975 (0.6)	2,132 (0.6)	1,445 (0.4)	562 (0.2)	
OR (95% CI)*	1.12 (1.01-1.25)	1.22 (1.11-1.35)	1.37 (1.22-1.53)	1.36 (1.13-1.63)	<.0001
Sessile serrated polyps					
No. of cases (%)	43 (0.1)	47 (0.1)	28 (0.0)	5 (0.0)	
No. of controls (%)	164 (0.0)	157 (0.0)	85 (0.0)	31 (0.0)	
OR (95% CI)*	1.06 (0.75-1.51)	1.39 (0.98-1.96)	1.68 (1.08-2.61)	0.80 (0.30-2.16)	0.02
Tubular adenomas					
No. of cases (%)	1,047 (1.5)	791 (1.2)	422 (0.6)	198 (0.3)	
No. of controls (%)	3,591 (1.1)	2,388 (0.7)	1,361 (0.4)	443 (0.1)	
OR (95% CI)*	1.24 (1.16-1.34)	1.51 (1.39-1.65)	1.40 (1.24-1.57)	1.94 (1.62-2.32)	<.0001
Tubulovillous adenomas					
No. of cases (%)	908 (1.3)	537 (0.8)	313 (0.5)	98 (0.1)	
No. of controls (%)	2,894 (0.9)	1,500 (0.4)	844 (0.3)	199 (0.1)	
OR (95% CI)*	1.32 (1.22-1.43)	1.50 (1.35-1.66)	1.58 (1.38-1.82)	2.22 (1.71-2.87)	<.0001
Villous adenomas					
No. of cases (%)	136 (0.2)	65 (0.1)	33 (0.0)	18 (0.0)	
No. of controls (%)	437 (0.1)	153 (0.0)	77 (0.0)	30 (0.0)	
OR (95% CI)*	1.18 (0.96-1.44)	1.69 (1.24-2.30)	1.84 (1.20-2.81)	2.06 (1.09-3.91)	<.0001

Abbreviations: CI, confidence interval; FDR, first-degree relative; OR, odds ratio.
*Multivariable conditional logistic regression was used to account for the matching on age, sex, year of birth, and county of residence, and was further adjusted for the number of FDRs with a history of CRC (continuous), year of birth (continuous), family size (continuous), income levels (quintiles), education (9 years or less, 10-12 years, >12 years, missing), total number of prior clinic visits (quintiles), number of prior endoscopies (0, 1, 2, ≥3), Charlson comorbidity score (continuous), and major comorbidities (all binary, including diabetes, cardiovascular disease, non-colorectal cancer, liver disease, chronic pulmonary disease, connective tissue disease, and peptic ulcer disease).

Supplementary Table 4. Association between family history of polyps in FDRs and risk of colorectal cancer according to cancer subsites*

No. of FDRs with different polyp subtypes	Proximal colon cancer		Distal colon cancer		Rectal cancer		$P_{heterogeneity}$ by cancer subsite‡
	No. of cases	OR (95% CI)†	No. of cases	OR (95% CI)†	No. of cases	OR (95% CI)†	
Any polyps							
0	4,999	1 (Ref)	6,014	1 (Ref)	21,984	1 (Ref)	
1	443	1.39 (1.23-1.58)	564	1.40 (1.26-1.57)	1,804	1.39 (1.31-1.47)	
≥2	49	1.85 (1.23-2.77)	60	1.68 (1.20-2.35)	169	1.79 (1.48-2.16)	
P for trend		<.0001		<.0001		<.0001	0.99
Conventional adenomas							
0	5,126	1 (Ref)	6,178	1 (Ref)	22,472	1 (Ref)	
1	340	1.47 (1.27-1.70)	426	1.41 (1.24-1.59)	1,392	1.47 (1.38-1.57)	
≥2	25	1.78 (1.02-3.12)	34	1.69 (1.09-2.64)	93	1.82 (1.41-2.34)	
P for trend		<.0001		<.0001		<.0001	0.77
Serrated polyps							
0	5,320	1 (Ref)	6,445	1 (Ref)	23,385	1 (Ref)	
1	160	1.23 (1.01-1.50)	186	1.31 (1.10-1.57)	553	1.21 (1.09-1.33)	
≥2	11	2.53 (1.06-6.03)	7	0.96 (0.37-2.46)	19	1.14 (0.68-1.92)	
P for trend		0.007		0.006		0.0003	0.67

Abbreviations: CI, confidence interval; FDR, first-degree relative; OR, odds ratio.

*Colon cases with no specified sublocation (n=32,616, 47.0%) were not included in the analysis.

†Multivariable conditional logistic regression was used to account for the matching on age, sex, year of birth, and county of residence, and was further adjusted for the number of FDRs with a history of CRC (continuous), year of birth (continuous), family size (continuous), income levels (quintiles), education (9 years or less, 10-12 years, >12 years, missing), total number of prior clinic visits (quintiles), number of prior endoscopies (0, 1, 2, ≥3), Charlson comorbidity score (continuous), and major comorbidities (all binary, including diabetes, cardiovascular disease, non-colorectal cancer, liver disease, chronic pulmonary disease, connective tissue disease, and peptic ulcer disease).

‡$P_{heterogeneity}$ was calculated using the contrast test method.

Supplementary Table 5. Stratified association between family history of any polyps in FDRs and risk of colorectal cancer (CRC)

	No. of FDRs with any polyps			P_{trend}	$P_{interaction}$
	0	1	≥2		
By sex					0.94
Men					
No. of cases (%)	33,474 (91.5)	2,830 (7.7)	292 (0.8)		
OR (95% CI)*	1 (Ref)	1.30 (1.24-1.36)	1.59 (1.37-1.85)	<.0001	
Women					
No. of cases (%)	28,844 (91.7)	2,390 (7.6)	230 (0.7)		
OR (95% CI)*	1 (Ref)	1.33 (1.27-1.41)	1.48 (1.25-1.75)	<.0001	
By relationship of FDRs					0.04
Parents					
No. of cases (%)	64,913 (95.4)	3,062 (4.5)	85 (0.1)		
OR (95% CI)*	1 (Ref)	1.35 (1.29-1.41)	1.32 (1.02-1.72)	<.0001	
Sibling					
No. of cases (%)	65,231 (95.8)	2,593 (3.8)	236 (0.3)		
OR (95% CI)*	1 (Ref)	1.27 (1.21-1.33)	1.39 (1.18-1.64)	<.0001	
By year of birth					0.01
<1940					
No. of cases (%)	26,750 (94.7)	1,367 (4.8)	117 (0.4)		
OR (95% CI)*	1 (Ref)	1.32 (1.24-1.41)	1.27 (1.01-1.59)	<.0001	
≥1940, <1950					
No. of cases (%)	23,062 (90.3)	2,224 (8.7)	243 (1.0)		
OR (95% CI)*	1 (Ref)	1.28 (1.21-1.35)	1.60 (1.36-1.88)	<.0001	
≥1950					
No. of cases (%)	12,506 (87.5)	1,629 (11.4)	162 (1.1)		
OR (95% CI)*	1 (Ref)	1.34 (1.26-1.43)	1.72 (1.40-2.12)	<.0001	
By year of CRC diagnosis					0.003†
<2008					
No. of cases (%)	29,957 (94.6)	1,592 (5.0)	116 (0.4)		
OR (95% CI)*	1 (Ref)	1.46 (1.37-1.56)	2.22 (1.73-2.86)	<.0001	
≥2008, <2012					
No. of cases (%)	14,186 (90.4)	1,382 (8.8)	128 (0.8)		
OR (95% CI)*	1 (Ref)	1.34 (1.25-1.44)	1.53 (1.22-1.91)	<.0001	
≥2012					
No. of cases (%)	18,175 (87.8)	2,246 (10.9)	278 (1.3)		
OR (95% CI)*	1 (Ref)	1.32 (1.25-1.40)	1.56 (1.34-1.82)	<.0001	

Abbreviations: CI, confidence interval; FDR, first-degree relative; OR, odds ratio.
*Multivariable conditional logistic regression was used to account for the matching on age, sex, year of birth, and county of residence, and was further adjusted for the number of FDRs with a history of CRC (continuous), year of birth (continuous), family size (continuous), income levels (quintiles), education (9 years or less, 10-12 years, >12 years, missing), total number of prior clinic visits (quintiles), number of prior endoscopies (0, 1, 2, ≥3), Charlson comorbidity score (continuous), and

major comorbidities (all binary, including diabetes, cardiovascular disease, non-colorectal cancer, liver disease, chronic pulmonary disease, connective tissue disease, and peptic ulcer disease).

†This is $P_{heterogeneity}$ calculated using the contrast test method.

Supplementary Table 6. Joint association of history of polyps and colorectal cancer (CRC) in first-degree relatives (FDRs) with risk of overall CRC and early-onset CRC (diagnosed before the age of 50 years).

	History of polyps in FDRs	History of CRC in FDRs	No. of cases (%)	No. of controls (%)	OR (95% CI)*	$P_{interaction}$
Overall CRC						
By number of FDRs with polyps	0	0	56,997 (83.7)	298,097 (89.3)	1 (Ref)	0.0005
	1	0	3,254 (4.8)	12,627 (3.8)	1.43 (1.38-1.50)	
	≥2	0	221 (0.3)	676 (0.2)	1.79 (1.52-2.10)	
	0	1	5,082 (7.5)	16,361 (4.9)	1.70 (1.65-1.76)	
	1	1	1,775 (2.6)	4,651 (1.4)	2.16 (2.04-2.29)	
	≥2	1	202 (0.3)	434 (0.1)	2.68 (2.24-3.20)	
	0	≥2	239 (0.4)	435 (0.1)	3.14 (2.65-3.71)	
	1	≥2	191 (0.3)	349 (0.1)	3.15 (2.61-3.81)	
	≥2	≥2	99 (0.1)	123 (0.0)	5.00 (3.77-6.63)	
By the youngest age at polyp diagnosis	No	No	56,997 (83.7)	298,097 (89.3)	1 (Ref)	0.001
	≥60 years	No	2,504 (3.7)	9,789 (2.9)	1.42 (1.35-1.48)	
	<60 years	No	971 (1.4)	3,514 (1.1)	1.55 (1.44-1.67)	
	No	≥60 years	4,126 (6.1)	13,896 (4.2)	1.63 (1.57-1.70)	
	≥60 years	≥60 years	1,522 (2.2)	4,097 (1.2)	2.12 (1.99-2.25)	
	<60 years	≥60 years	224 (0.3)	477 (0.1)	2.70 (2.28-3.20)	
	No	<60 years	1,195 (1.8)	2,900 (0.9)	2.24 (2.08-2.40)	
	≥60 years	<60 years	193 (0.3)	340 (0.1)	3.22 (2.67-3.89)	
	<60 years	<60 years	328 (0.5)	643 (0.2)	2.85 (2.48-3.29)	
Early-onset CRC						
By number of FDRs with polyps	0	0	6,295 (85.4)	34,095 (93.0)	1 (Ref)	0.001
	1	0	368 (5.0)	1,154 (3.1)	1.82 (1.59-2.08)	
	≥2	0	20 (0.3)	29 (0.1)	3.97 (2.09-7.53)	
	0	1	459 (6.2)	1,004 (2.7)	2.67 (2.36-3.03)	
	1	1	170 (2.3)	340 (0.9)	3.03 (2.46-3.73)	
	≥2	1	21 (0.3)	17 (0.0)	9.83 (4.63-20.90)	
	0	≥2	22 (0.3)	10 (0.0)	12.10 (5.30-27.60)	
	1	≥2	6 (0.1)	15 (0.0)	2.60 (0.92-7.35)	
	≥2	≥2	11 (0.1)	4 (0.0)	16.57 (4.81-57.13)	
By the youngest age at polyp diagnosis	No	No	6,295 (85.4)	34,095 (93.0)	1 (Ref)	0.29
	≥60 years	No	229 (3.1)	752 (2.1)	1.76 (1.49-2.08)	
	<60 years	No	159 (2.2)	431 (1.2)	2.01 (1.64-2.46)	
	No	≥60 years	241 (3.3)	680 (1.9)	2.02 (1.72-2.38)	
	≥60 years	≥60 years	104 (1.4)	272 (0.7)	2.26 (1.76-2.91)	
	<60 years	≥60 years	8 (0.1)	23 (0.1)	3.30 (1.40-7.76)	
	No	<60 years	240 (3.3)	334 (0.9)	4.31 (3.58-5.19)	
	≥60 years	<60 years	17 (0.2)	19 (0.1)	6.08 (2.94-12.60)	
	<60 years	<60 years	79 (1.1)	62 (0.2)	7.90 (5.39-11.57)	

Abbreviations: CI, confidence interval; FDR, first-degree relative; OR, odds ratio.
*Multivariable conditional logistic regression was used to account for the matching on age, sex, year of birth, and county of residence, and was further adjusted for year of birth (continuous),

family size (continuous), income levels (quintiles), education (9 years or less, 10-12 years, >12 years, missing), total number of prior clinic visits (quintiles), number of prior endoscopies (0, 1, 2, ≥3), Charlson comorbidity score (continuous), and major comorbidities (all binary, including diabetes, cardiovascular disease, non-colorectal cancer, liver disease, chronic pulmonary disease, connective tissue disease, and peptic ulcer disease).

Supplementary Table 7. Summary of results of sensitivity analyses for assessing the association between family history of any polyps and risk of colorectal cancer (CRC)

Analysis	Cases, n (%)	Controls, n (%)	Multivariable-adjusted OR (95% CI)*	Multivariable + FH of CRC-adjusted OR (95% CI) †
Using mortality of incidence CRC as the study outcome				
Family history of any polyps				
No	17,803 (93.0)	89,697 (95.1)	1 (Ref)	1 (Ref)
Yes	1,345 (7.0)	4,605 (4.9)	1.61 (1.50-1.73)	1.40 (1.30-1.51)
No. of FDRs with any polyps				
0	17,803 (93.0)	89,697 (95.1)	1 (Ref)	1 (Ref)
1	1,236 (6.5)	4,333 (4.6)	1.58 (1.47-1.70)	1.39 (1.29-1.50)
≥2	109 (0.6)	272 (0.3)	2.05 (1.59-2.66)	1.55 (1.19-2.02)
P_{trend}			<.0001	<.0001
Restricted to CRC cases diagnosed after 2008 and family history assessment to post-2008 in Stockholm only				
Family history of any polyps				
No	36,132 (99.3)	175,711 (99.5)	1 (Ref)	1 (Ref)
Yes	263 (0.7)	885 (0.5)	1.41 (1.22-1.62)	1.24 (1.07-1.43)
No. of FDRs with any polyps				
0	36,132 (99.3)	175,711 (99.5)	1 (Ref)	1 (Ref)
1	255 (0.7)	870 (0.5)	1.39 (1.21-1.61)	1.23 (1.06-1.42)
≥2	8 (0.0)	15 (0.0)	2.09 (0.86-5.07)	1.67 (0.69-4.07)
P_{trend}			<.0001	<.0001

Abbreviations: CI, confidence interval; FDR, first-degree relative; FH, family history; OR, odds ratio.

*Multivariable conditional logistic regression was used to account for the matching on age, sex, year of birth, and county of residence, and was further adjusted for year of birth (continuous), family size (continuous), income levels (quintiles), education (9 years or less, 10-12 years, >12 years, missing), total number of prior clinic visits (quintiles), number of prior endoscopies (0, 1, 2, ≥3), Charlson comorbidity score (continuous), and major comorbidities (all binary, including diabetes, cardiovascular disease, non-colorectal cancer, liver disease, chronic pulmonary disease, connective tissue disease, and peptic ulcer disease).

†Further adjusted for the number of FDRs with a history of CRC (continuous).

Supplementary Table 8. Prevalence of common diseases diagnosed in individuals with and without a family history of polyps among the control group

Individual comorbidity, n (%)	Negative family history of polyps (n=314,893)	Positive family history of polyps (n= 18,860)
Myocardial Infarction	15,931 (4.2)	941 (3.8)
Congestive Heart Failure	8,101 (2.2)	485 (2.0)
Peripheral Vascular Disease	6,369 (1.7)	464 (1.9)
Cerebrovascular Disease	19,581 (5.2)	1,307 (5.3)
Dementia	2,481 (0.7)	149 (0.6)
Chronic Pulmonary Disease	18,462 (4.9)	1,415 (5.8)
Connective Tissue Disease-Rheumatic Disease	6,691 (1.8)	490 (2.0)
Peptic Ulcer Disease	4,629 (1.2)	364 (1.5)
Mild Liver Disease	2,425 (0.6)	181 (0.7)
Diabetes without complications	17,870 (4.7)	1,207 (4.9)
Diabetes with complications	6,575 (1.7)	446 (1.8)
Paraplegia and Hemiplegia	1,466 (0.4)	124 (0.5)
Renal Disease	2,487 (0.7)	169 (0.7)
Cancer*	30,789 (8.2)	2,435 (9.9)
Moderate or Severe Liver Disease	693 (0.2)	54 (0.2)
Metastatic Carcinoma of Unspecified Sites	3,665 (1.0)	297 (1.2)
AIDS/HIV	168 (0.0)	6 (0.0)

*Excluding colorectal cancer.
